# Open Online Courses for Informal Carers: Systematic Integrative Review

**DOI:** 10.2196/72808

**Published:** 2025-08-28

**Authors:** Kalya Win Aung, Angela Kibia, Juliana Onwumere

**Affiliations:** 1 Department of Psychology, Institute of Psychology, Psychiatry and Neuroscience, King's College London, Henry Wellcome Building, De Crespigny Park, London, SE5 8AF, United Kingdom, 44 7386692555

**Keywords:** carers, caregivers, massive open online course, MOOC, open online course, families, online learning

## Abstract

**Background:**

Informal carers, people providing unpaid support to relatives or close others with an illness, disability, or advanced age-related care needs, are key stakeholders in health care systems. Carers have their own health and well-being challenges; however, their needs and care pathways are often overlooked by health care providers. Open online courses offer opportunities to address the information and support needs of large numbers of carers. However, our collective understanding of the design and outcomes of courses and learner experiences is limited.

**Objective:**

This systematic integrative review aimed to map the characteristics of open online courses for informal carers, explore learner experiences, and identify barriers and enablers to participation to inform the design of future courses.

**Methods:**

Following PRISMA (Preferred Reporting Items for Systematic Reviews and Meta-Analyses) guidelines, we systematically searched 4 electronic databases (APA PsycINFO, CINAHL, EMBASE, and MEDLINE) for papers published from inception to January 30, 2025. Included papers were peer-reviewed, in the English language, and reported on the development, delivery, or outcomes of open online courses for informal carers aged more than 16 years. Excluded papers had no carer focus or were conference abstracts. Two reviewers independently screened titles and abstracts for eligibility. Backward and forward citation searches were conducted. Due to study heterogeneity, data on paper methodology, course characteristics, and course evaluations were extracted and synthesized narratively. Quality assessments of quantitative papers and the quantitative components of mixed methods papers used the Effective Public Health Practice Project (EPHPP) Quality Assessment Tool. Qualitative components within mixed methods papers were appraised using the Critical Appraisal Skills Program (CASP) toolkit.

**Results:**

Searches identified 201 papers, of which 10 (6 quantitative and 4 mixed methods) met the inclusion criteria. No qualitative papers were identified. All included courses were massive open online courses. Sample sizes ranged from 3 to 17,591 participants, primarily targeting carers of older individuals (n=4). Completion rates ranged from 42% to 67% (n=5). Five papers reported improved carer knowledge and application of skills. Key enablers to learning included course accessibility and flexibility. Key barriers to learning included limited peer interaction, technical difficulties, time constraints, language challenges, and online privacy challenges. Most papers were of weak quality, except for 1 strong quantitative RCT.

**Conclusions:**

The evidence was limited by moderate-to-weak study quality, inconsistent measures, and exclusion of gray literature. Despite these limitations, findings suggest that open online courses may improve carers’ knowledge and skills and enable accessible, flexible learning. However, barriers to learning, including limited learner-to-learner interaction within and external to the course, time constraints, and digital or language barriers, highlight the need for more inclusive and interactive course designs. Further high-quality research is needed to standardize outcomes.

## Introduction

The population aged 60 years and older is projected to grow from 1.2 billion in 2024 to 2.1 billion by 2050, accounting for 26% of the global population [1]. This demographic shift is expected to increase pressure on health and social care systems as more individuals experience age-related diseases and multimorbidities, leading to a greater need for and reliance on informal care [2]. Though there is no uniform and uniformly accepted definition, informal carers are commonly understood as those who provide unpaid care and support to relatives or significant others living with long-term health conditions, disability, or care needs presenting in older adult years [3]. They play a crucial role in sustaining health care systems [4] and contribute an estimated 16.4 billion hours of unpaid care each day, the equivalent of 2 billion people working 8-hour shifts without compensation [5]. While informal care spans all age groups and conditions, the need for support from informal carers is expected to increase as populations continue to age [5].

Although informal care provides significant societal and economic contributions, it can have negative impacts on the physical and mental health of the informal carer [6]. Compared to noncarer peers, evidence confirms that informal carers experience lower levels of well-being [7,8], higher rates of common mental disorders, such as depression and anxiety [9,10], greater sleep disturbances [11,12], and an overall poorer quality of life [13]. Illness-related demands, including uncertainty about the illness timeline and course and a deterioration in the care recipient’s needs, can contribute to carers’ poorer well-being. In turn, this reduced well-being can reduce their capacity to provide care and hinder their care recipient’s well-being and outcomes [14]. Despite a well-established relationship between informal carer status and poorer carer health, health and social care providers often prioritize the care recipient’s needs over those of the carer, leaving informal carers feeling overlooked or abandoned [15]. Addressing these issues, especially against the backdrop of an aging global population, requires better support structures for informal carers.

According to the International Telecommunication Union, an estimated 68% of the global population had access to the internet in 2024, including 92% of households in the United States, 98% in the United Kingdom, and 74% in China [16]. As more informal carers turn to the internet for assistance [17], digital tools are becoming increasingly valuable in supporting their caring roles. Digital technologies encompass a variety of formats, including mobile apps [18] and web-based platforms [19], which provide valuable information, resources, and training related to care. They can raise awareness of the issues carers face and can also enhance their understanding of the needs of those they care for, offer insights into adaptive coping strategies, and build supportive communities.

In the last decade, open online courses have gained attention for their potential to deliver targeted, equitable, and supportive education to disadvantaged groups [20], including informal carers. Open online courses are characterized as such because they are (1) free to enroll (“open”), (2) offered via the internet (“online”), and (3) designed around structured learning objectives (“courses”) [21]. They typically include features, such as short video lectures, automated assessments that give instant feedback to test understanding, and online discussion forums where participants can ask questions and share ideas, often with guidance from course instructors. Open online courses encompass various formats designed to deliver structured learning in an accessible, flexible manner. For example, corporate open online courses are made specifically for organizations, small open online courses cater to specialized topics with smaller audiences, and nano open online courses require less than 20 hours of engagement. Among the variants, massive open online courses (MOOCs) are the most prominent, distinguished by their ability to reach thousands of learners worldwide simultaneously, unlike the smaller-scale reach of other open online courses. By the end of 2020, MOOCs had attracted over 180 million learner enrollments across more than 16,300 courses globally [22]. Their scalability allows them to overcome traditional educational barriers, such as geographical and financial constraints, and broaden global access to learning opportunities [23].

Despite their popularity, evidence on the effectiveness and impact of open online courses for informal carers is limited. While several reviews have investigated the application of open online courses in health education [24-26], to date, there have been no reviews that have focused on open online courses for informal carers. Lamura et al [27,28] highlight the potential of novel technological solutions to empower and support carers. However, it remains unclear whether challenges associated with existing open online courses, such as high dropout rates [29], difficulty with navigating course platforms [30], and variable levels of digital literacy in learners [31], also affect those designed for informal carers.

To address the evidence gaps, this systematic integrative review seeks to map the key characteristics of open online courses designed for informal carers and learners’ experiences. In addition, the review will identify common barriers and enablers to participation and deployment of these open online courses, aiming to provide recommendations that guide the future development, implementation, and assessment of these resources for supporting informal carers.

## Methods

### Overview

The review followed Whittemore and Knafl’s [32] integrative review methodology and current PRISMA (Preferred Reporting Items for Systematic Reviews and Meta-Analyses) guidelines (Checklist 1; [33]). A protocol was registered on the International Prospective Register of Systematic Reviews (CRD42024532766). This allowed for the synthesis of both empirical (quantitative and qualitative) evidence and theoretical literature related to open online courses for informal carers.

### Eligibility Criteria

The review used the population, intervention, comparator, outcome (PICO) framework for inclusion in the study [34]:

1. Population: Learners aged 16 years old and above from any geographic area who participated in an open online course designed for informal carers.
2. Intervention: Open online courses designed for informal carers. This includes studies that reported on course development, or delivery, or learner outcomes using qualitative, quantitative, or mixed methods. In accordance with Tashakkori and Creswell [35], papers were classified as mixed methods if they combined both qualitative and quantitative approaches or methods within the same study.
3. Comparator: Papers did not need to include a comparator for inclusion in this systematic integrative review.
4. Outcome: Learner-focused outcomes, such as knowledge, skills, perceived support, attitudes, course satisfaction, and feedback on course aspects were assessed.

In addition to the PICO framework, the following criteria (Textbox 1) were used.

Textbox 1. **Inclusion and exclusion criteria.**

**Inclusion criteria**

- Studies published in English.
- Studies published in peer-reviewed journals.

**Exclusion criteria**

- Studies on e-learning (ie, learning on the internet) without a specific focus on open online courses.
- Studies on open online courses that did not mention informal carers as a target learner in their course title or description.
- Conference abstracts, opinion pieces, unpublished dissertations, non-peer-reviewed papers, or book chapters.

### Search Strategy

The search strategy was developed by the first author (KWA) in consultation with an academic librarian specializing in health sciences. Keywords were informed by the research question and relevant literature on informal carers and internet-based education. A formal search was conducted on July 18, 2024, by KWA, who searched the following databases: (1) APA PsycINFO, (2) CINAHL, (3) EMBASE, and (4) MEDLINE. The following search string was used: (“informal” or “family” or “unpaid”) AND (“carers” or “caregivers” or “care” or “caring”) AND “online course.” This combination of terms was chosen to capture the varied terminology used to describe informal carers across papers and was adapted to match the requirements of each database. There were no date or demographic restrictions on the included studies. The complete search strategy for each database can be found in Multimedia Appendix 1. Backward and forward citation was also conducted on the reference lists of included studies. The search was rerun on 30 January, 2025, to ensure inclusion of the most recent research.

### Selection of Studies

All papers retrieved from database searches were imported into EndNote 21 (Clarivate; [36]), and duplicates were removed. The title and abstract of each paper were imported into Covidence software (Veritas Health Innovation [37]) and independently screened for eligibility by 2 reviewers (KWA and JO). Any studies that appeared to meet the eligibility criteria, or where eligibility was unclear, proceeded to the next stage of full-text screening. Full texts were then assessed independently by the same 2 reviewers against inclusion and exclusion criteria. Reasons for exclusion at this stage were recorded in Covidence. Disagreements between the 2 reviewers were resolved by discussion and reaching consensus.

### Data Extraction

A structured summary table was developed based on the research question and objectives and piloted on 2 papers to ensure consistency. The extracted information included key paper characteristics (ie, author, publication year, origin, sample size, sample characteristics, and study design), open online course characteristics (ie, course type, duration, topic areas, development, and pedagogical approaches), and evaluation details (ie, evaluation methods, learning outcomes, barriers and enablers to learning, and course feedback). Data were extracted by KWA and reviewed by JO for accuracy.

### Assessment of Methodological Quality

The methodological quality of selected papers was reviewed. Quantitative papers were critically appraised using the Effective Public Health Practice Project (EPHPP) Quality Assessment Tool [38]. This tool, originally designed for evaluating health and medical literature, has demonstrated content and construct validity [38,39]. The EPHPP tool assesses quantitative research based on 8 key criteria: selection bias, study design, confounders, blinding, data collection methods, withdrawals and dropouts, intervention integrity, and analyses. Each criterion is scored on a 3-point scale. The global study rating is classified as “strong” if no components are rated as weak, “moderate” if there is 1 weak rating, and “weak” if there are 2 or more weak ratings.

The Critical Appraisal Skills Program (CASP) toolkit [40] was applied to qualitative papers. This is the preferred tool for assessing the methodological quality of qualitative studies in health care research and has been used in reviews related to the care of conditions like dementia [41-43]. The toolkit assesses the applicability, reliability, and validity of qualitative research through 10 questions. These questions cover the research aim, methodology, research design, recruitment strategy, data collection, relationships between researcher and participants, ethical considerations, data analysis, findings, and the research value. Studies obtaining 8 or more “yes” ratings are classified as “strong”; 5-7 “yes” ratings are classified as “moderate,” and fewer than 5 “yes” ratings are defined as “weak.”

For mixed methods studies, both the EPHPP and CASP were used to appraise the respective qualitative and quantitative components. This approach enabled a more rigorous, design-specific appraisal and maintained consistency with the evaluation of methodologically singular studies. This combination of appraisal tools has also been adopted in other systematic reviews of carer and health-related interventions [41,44].

Quality assessment was conducted independently by 2 reviewers (KWA and AK - a master’s level researcher). Both reviewers were blinded to each other’s decisions. The 2 raters’ respective results were compared, and any discrepancies were resolved through discussion and consultation with a third reviewer (JO) to establish a consensus.

### Data Synthesis

Narrative synthesis allows for the inclusion of qualitative, quantitative, and mixed methods studies to be reported in a systematic manner [45]. Given the heterogeneity of methodologies and outcome measures in the included studies, a narrative synthesis was deemed the most suitable option for this review. Guided by the framework outlined by Popay et al [45], the synthesis was conducted by KWA, who first identified similarities and differences between the included studies and their measured outcomes. This enabled the categorization of findings according to the characteristics of each open online course and the experience of learning on each course. Categories were further refined through discussion and consultation with JO. This resulted in a preliminary synthesis of the studies, which explored recurring patterns and relationships throughout the data.

## Results

### Search Process

**Figure 1.**
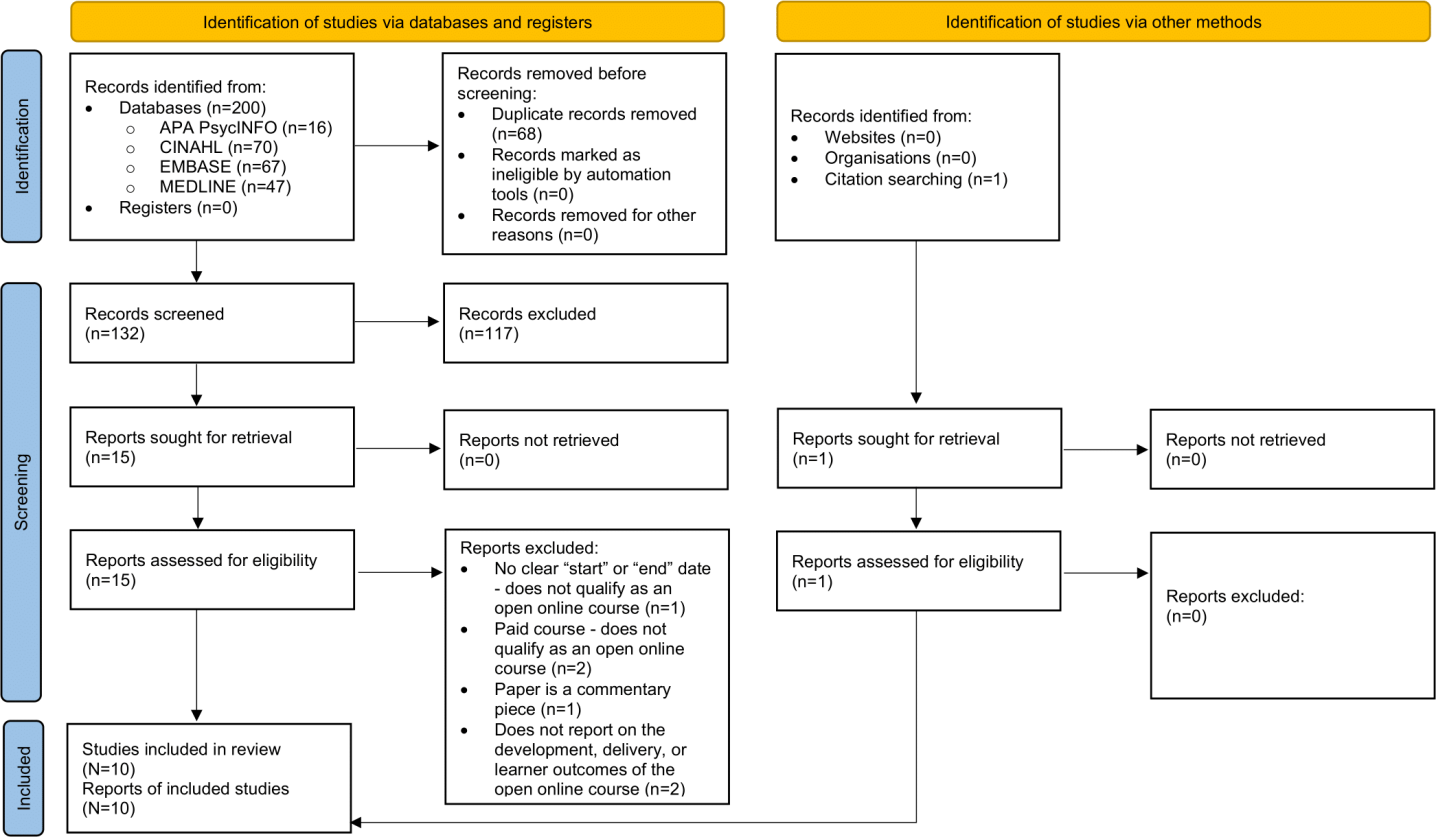
Preferred Reporting Items for Systematic Reviews and Meta-Analyses flow diagram of literature search and screening results.

A PRISMA diagram of the literature search and screening process is shown in Figure 1. The initial and rerun database searches yielded 200 records in total, with 1 additional record identified through a backward citation search. After removing duplicates, 132 records remained for screening, of which 117 records were excluded. Fifteen records were deemed eligible for full-text review. Of these, 6 records were excluded for not meeting inclusion criteria: 2 papers’ [46,47] courses were not free to enroll and therefore were not considered to be open online courses; 1 paper’s [48] course did not have a set start and end date so it was not classified as an open online course; another paper was excluded as it was a commentary piece [49]; and 2 papers [50,51] were excluded for not reporting on the development, delivery, or learner outcomes of the open online course. Ten records [52-61] were included in the final review.

### Paper Characteristics

The 10 identified papers [52-61] included in this systematic integrative review came from 9 studies [52-60]. They comprised 6 quantitative papers [54,56-58,60,61] and 4 mixed methods papers [52,53,55,59]. Among the quantitative papers, there were 4 cohort studies [54,56-58], 1 randomized controlled trial (RCT) [60], and a protocol for the same RCT [61]. The mixed methods papers included 3 cohort studies [52,53,59] and 1 case study [55]. Notably, 9 [52-55,57-61] of the 10 papers [52-61] were published from the year 2020 onwards. The papers originated from several countries, including Australia [53,54,56,57,60,61], Brazil [55], Canada [59], Portugal [58], and the United Kingdom [52]. A detailed summary of the characteristics of the included papers is presented in Tables1^3^.

**Table 1.** Country of origin, design, and sample size range.

Authors	Origin	Design	Sample size range
Blakemore et al [Bibr R52][52]	United Kingdom	Mixed Methods	14-909
Borchard et al [53][Bibr R53]	Australia	Mixed Methods	4712
Claflin et al [Bibr R54][54]	Australia	Quantitative	1549-3518
do Canto et al [Bibr R55][55]	Brazil	Mixed Methods	3
Eccleston et al [Bibr R56][56]	Australia	Quantitative	4894
Fair et al [Bibr R57][57]	Australia	Quantitative	17,591
Lumini et al [Bibr R58][58]	Portugal	Quantitative	11‐33
Rottenberg and Williams [Bibr R59][59]	Canada	Mixed Methods	5-111
Whittingham et al [Bibr R61][61]	Australia	Quantitative Protocol	Aiming for 66
Whittingham et al [Bibr R60][60]	Australia	Quantitative	67

a %F: percentage female.

b %BD: percentage with a bachelor’s degree.

**Table 2.** Sample characteristics

Target group	Age (years)	Female (%)	Bachelor’s degree (%)
Cancer genomics community	—	—	—
Informal carers of dementia patients and noncarers	Median 50 (IQR 35-61)	82	25
Multiple sclerosis community	Mean 44 (SD 13)	84	59
Informal carers of older stroke survivors	Mean 53	—	—
Dementia community	Median 39 (IQR 29-48)	90	32
Traumatic brain injury community	Mean 43 (SD 15)	83	36
Informal carers during COVID-19	Mean 54 (SD 9)	73	30
Informal carers of older adults	Mode 45‐64	72	—
Parents of children with cerebral palsy	—	—	—
Parents of children with cerebral palsy	Mean 38-40 (SD 6-9)	91-100	44-59

**Table 3.** Course characteristics, development, evaluation methods, and learner outcomes.

Authors	Course characteristics (subject, learning platform, developer, duration, and number of modules)	Development process	Evaluation methods	Learner outcomes
Blakemore et al [52][Bibr R52]	MOOC^a^ on cancer genomics (platform: FutureLearn, developer: Global University Systems, duration: 6 week, modules: NR^b^)	Not recorded	Written summary assignment Digital literacy task Learner comments	Improved digital literacy Engaging but limited varied learning methods
Borchard et al [53][Bibr R53]	MOOC on dementia (platform: Wicking Dementia Center, developer: University of Tasmania, duration: 7 week, modules: 3)	Not recorded	Engagement metrics (posts and replies in discussion boards)	Higher engagement among carers Barriers included technical issues and accessibility challenges
Claflin et al [Bibr R54][54]	MOOC on multiple sclerosis (platform: Menzies Institute, developer: University of Tasmania, duration: 6 week, modules: 6)	Modeled after the Wicking Dementia Research and Education Centre MOOCs Iterative updates	Learning management system data Feedback surveys	High satisfaction Age and education influenced outcomes
do Canto et al [55][Bibr R55]	MOOC for informal carers of older stroke survivors (platform: Moodle, developer: Moodle HQ, duration: NR, modules: 12)	Developed by nurses, with iterative expert input on content analysis, storyboarding, digital creation, platform integration, and preliminary evaluation	Assessment instrument Preliminary course feedback	Positive feedback on accessibility and content Suggestions to enhance audio
Eccleston et al [56][Bibr R56]	MOOC on dementia (platform: Wicking Dementia Center, developer: University of Tasmania, duration: 9 week, modules: NR)	Not recorded	Pre- and postcourse assessments Knowledge tests	Significant knowledge increase Completion rate exceeded average MOOCs
Fair et al [Bibr R57][57]	MOOC on traumatic brain injury (platform: Wicking Dementia Center, developer: University of Tasmania, duration: 7 week, modules: 4)	Modeled after the Wicking Dementia Research and Education Center MOOCs Iterative updates Piloted before launch	Learning management system data Precourse survey	TBI^c^ education was the main motivation for enrollment Higher completion in those with TBI experience and retirees Lower in educated individuals without TBI experience and carers
Lumini et al [Bibr R58][58]	MOOC for informal carers during COVID-19 (platform: NAU, developer: FCCN Unit of the Foundation for Science and Technology, duration: NR; modules: 9)	Evidence-based Plain language Delphi-validated by experts	Pre- and postknowledge questionnaires Technology Acceptance Model	Increased knowledge and self-confidence Challenges balancing time and providing care
Rottenberg and Williams [Bibr R59][59]	MOOC for informal carers of older adults (platform: Desire2Learn Open Courses, developer: Desire2Learn, duration: 8 week, modules: 4)	Codeveloped by experts across McMaster research groups	Pre- and postcourse surveys Telephone interviews Focus groups	Flexible and accessible Suggestions for better navigation and engagement
Whittingham et al [Bibr R61][61]	MOOC on parenting for children with cerebral palsy (platform: edX, developer: 2U, duration: 10 week, modules: NR)	Adapted from parenting and ACT^d^ techniques	Embedded course questions Likert scale feedback Qualitative comments (proposed evaluation)	Not recorded
Whittingham et al [Bibr R60][60]	MOOC on parenting for children with cerebral palsy (platform: edX, developer: 2U, duration: 10 week, modules: NR)	Adapted from parenting and ACT techniques	Intent-to-treat sample Multilevel modeling Full-information maximum likelihood	Improved parenting skills, maintained at 6-month follow-up Praised for flexibility and interactivity

a MOOC: massive open online course.

b NR: not recorded.

c TBI: traumatic brain injury.

d ACT: acceptance and commitment therapy.

### Sample Characteristics

Sample sizes in the included papers ranged from 3 [55] to 17,591 [57] participants. The papers targeted different informal carer populations. A total of 4 papers [53,55,56,59] focused on carers of older adults, including 2 papers [53,56] on carers of people living with dementia, 1 paper [59] on carers of older adults, and 1 paper [55] on carers of older individuals who survived strokes. Notably, 5 papers [52,54,57,60,61] had a focus on carers of other chronic conditions, including a protocol and subsequent study on parents of children with cerebral palsy [60,61], carers of individuals with multiple sclerosis [54], carers of individuals with cancer [52], and carers of individuals with traumatic brain injury [57]. In addition, 1 paper [58] addressed carers in the context of the COVID-19 pandemic.

Ages of the learners were reported in 7 [53-59] of the 10 papers [52-61]. Participants were typically middle-aged adults, with their mean ages ranging from 38 (SD 6) [60] to 54 (SD 9) years [58]. The gender (or sex) distribution of learners was reported in 7 papers [53,54,56-60], and in each, over 70% of participants were reported as female.

### Course Development

A total of 7 [54,55,57-61] of 10 papers [52-61] reviewed reported details about the development process of their open online course. In these papers, the process typically commenced with a needs assessment to identify the specific challenges faced by the target carer group. For example, Lumini et al [58] conducted a Delphi study with experts to select and validate care topics relevant to the COVID-19 pandemic, while Rottenberg and Williams [59] designed their course to meet informal carers’ needs for practical, accessible information on health care issues for older adults. Claflin et al [54] and Fair et al [57] developed their courses based on existing MOOCs for participants from diverse backgrounds (ie, the Wicking Dementia Research and Education Center MOOCs). Similarly, Whittingham et al [60,61] tailored their course based on already established Acceptance and Commitment Therapy techniques from a previously conducted RCT for parents of children with cerebral palsy, as well as relevant parenting literature. Do Canto et al [55], Fair et al [57], and Rottenberg and Williams [59] used pilot testing to refine course structure, content, and usability.

### Course Implementation

#### Content

The open online courses in the included papers were all presented as MOOCs, the most popular format of open online course, where several thousand learners can participate at the same time. The reported course content was tailored to address caring challenges for specific health conditions or caring situations. For example, courses targeted at carers of individuals with dementia focused on understanding the progression of dementia and managing related behavioral symptoms [53,56]. In contrast, courses targeting parents of children with cerebral palsy aimed at enhancing parenting skills and reducing carer stress [60,61]. One course on traumatic brain injury provided information on the condition’s impacts and how to manage caring for survivors [57]. Some courses were designed to address situational challenges, such as best practices for general care provision during the COVID-19 pandemic [58] and the essentials for family care of older persons [59] [48].

#### Duration

The length of course runs was variable. Overall, 8 [52-54,56,57,59-61] out of 10 papers [52-61] specified course length, where durations ranged from 6 weeks [52,54] to 10 weeks [60,61].

#### Delivery

The courses used a range of methods to deliver content to learners. A key feature across all courses was modular content, which allowed participants to flexibly engage with material and learn and progress at their own pace. Qualitative findings from Rottenberg and Williams [59] highlighted that carers valued the self-paced nature of the course, as the ability to “hop online anytime” allowed them to effectively balance care duties with course participation.

All but 2 courses [55,58] featured a discussion forum to facilitate peer interaction and encourage a community learning environment. All courses reported across the 10 papers [52-61] also included some form of supplementary material, such as quizzes [52-54,56-59], downloadable resources [55,57-59], or journaling activities [60,61], to reinforce learning and assess progress.

#### Accessibility

All 10 papers’ [52-61] courses were offered online and free to access. However, Blakemore et al [52] allowed learners to pay an optional fee to complete a written peer assessment.

Fair et al [57] noted that their MOOC on traumatic brain injury was only available in English but adhered to Web Content Accessibility Guidelines, a set of standards designed to make web-based content more accessible to people with disabilities, such as those with visual, auditory, or cognitive impairments. This MOOC also incorporated accessibility features, such as colorblind-friendly palettes, screen reader compatibility, subtitles, transcripts, and downloadable content summaries. The remaining 9 papers [52-56,58-61] did not specify if their open online courses were available in languages other than English, leaving the accessibility of learning for non-English speakers unclear. However, Lumini et al [58] highlighted their use of plain language methods when designing the course content, which summarized medical jargon into an easily understandable format for nonscientific audiences.

The web-based format of the courses removed most geographical and financial barriers to accessing support [55,59]. This was particularly beneficial for learners in remote or rural areas where resources were limited [59]. However, infrastructure challenges, such as lack of internet access in certain regions, remained a barrier for some learners, as noted in participant interviews from Rottenberg and Williams’ [59] paper.

### Course Engagement and Participation

#### Time Spent on Course

Learner engagement was assessed using different measures across the included studies. Two papers [54,55] used the average time spent on the course as an indicator of engagement. Claflin et al [54] reported that learners spent an average of 2.2 hours per week on their course about multiple sclerosis, while do Canto et al [55] reported that learners engaged for an average of 34 minutes across the 1-week preliminary evaluation phase of their course for carers of older stroke survivors.

#### Discussion Forum Interaction

Peer interaction through discussion forums was another metric used by 2 papers [53,59] to gauge learner engagement. Borchard et al [53] found that informal carers were more active on discussion forums, posting and replying significantly more often than noncarers. However, Rottenberg and Williams [59] highlighted that peer interaction in discussion boards was reduced by the self-paced nature of the course, which resulted in asynchronous participation. Learners who completed the course content earlier often found that the discussion boards were empty or lacked meaningful conversation because others had not yet reached the same material [59].

Privacy concerns related to sharing personal or sensitive information publicly on the web also discouraged some learners from discussion forum participation [59]. Quantitative survey data from Rottenberg and Williams [59] confirmed these challenges, with only 40% (14/35) of participants feeling comfortable sharing ideas in written format online and 43% (15/35) confident in using and contributing to online discussion groups when seeking help or information. The absence of consistent peer interaction left some learners feeling isolated, with 1 participant stating they did not find the *“*tribe*”* they were hoping for. In response, learners suggested incorporating videoconferencing to facilitate real-time communication, helping to create a stronger sense of community within the course.

#### Maintaining Engagement Over Course Runs

Two papers [52,57] collected longitudinal data about engagement on their respective courses. After running their cancer genomics MOOC 8 times over 5 years, Blakemore et al [52] found that the number of active learners (ie, learners who marked at least 1 step on the course as “complete”) decreased with each run. In the first run, 171 learners submitted assignments while only 17 learners did so in the eighth run. This decline was likely influenced by a change in the MOOC platform’s certification model, which restricted access to assignments to paid learners. Similarly, Fair et al’s [57] traumatic brain injury MOOC saw a drop in enrollments across iterations, from 9012 in June 2021 to 3249 in August 2022.

#### Navigational and Technical Barriers

Navigational and technical difficulties were identified as barriers to course engagement. While some learners found the platforms user-friendly [55,59], others struggled to navigate modules, access videos, or locate resources [59]. Although these challenges were more frequently reported by those who identified as older carers, even some technology-savvy participants described the platform as “clumsy.*”*

These barriers occasionally deterred continued participation, as some learners gave up after being unable to resolve technical issues. Access to technical support was crucial for maintaining engagement on the courses. In Rottenberg and Williams’ [59] MOOC, the availability of nursing students or other support personnel to assist with technical problems was highly valued. However, learners also noted the need for more immediate and accessible help options, underscoring the importance of technical support systems for maintaining engagement.

### Course Evaluation and Outcomes

#### Learner Outcomes

The included papers measured a range of learner-focused outcomes from participation in open online courses. Excluding the RCT protocol by Whittingham et al [61], which did not collect outcome data, 5 papers [52-54,56,58] reported significant improvements in learners’ knowledge about course content or care provision skills.

Beyond knowledge gains, 2 papers [54,60] observed additional positive outcomes following participation. Whittingham et al [60] reported that after completing the course, participants were more likely to seek support from friends and family, stay connected to others, and perceive their lives as meaningful and fulfilling. These positive changes were maintained at follow-up, 6 months after the intervention. However, Whittingham et al [60] did not find significant improvements in learners’ psychological adjustment (eg, their ability to cope with emotional challenges) or in overall well-being, which relates to general mental and emotional health, among carers.

Claflin et al [54] reported that almost two-thirds of course completers applied the course material in their daily lives by the end of the program. Carers, especially those without a university education, were the most likely to report applying the material (140/201, 69.65%) while noncarers with a university education were the least likely to do so (279/483, 57.76%).

Rottenberg and Williams [59] collected pre- and postcourse data assessing participants’ caring roles, technological access and usage, and experiences on the course. Quantitative data could not be compared given the large discrepancy between the number of completed precourse surveys (n=111) versus postcourse surveys (n=39). However, qualitative findings revealed that for carers who had been providing care for a longer time, sharing experiences and peer support on discussion forums was “really comforting in a lot of ways*”* [59].

Fair et al [57] did not collect postcourse outcome data. However, their precourse survey revealed that over 90% (8983/9710) of MOOC participants agreed or strongly agreed with enrollment reasons relating to increasing their knowledge of traumatic brain injury.

#### Satisfaction

Three papers [54,58,59] noted that overall satisfaction with the open online courses was high. Claflin et al [54] found that almost 97% (1502/1549) of participants were satisfied with the course. Similarly, Lumini et al [58] reported 81% (27/33) of participants were satisfied with the web-based format of the course, especially during the COVID-19 pandemic. Rottenberg and Williams [59] reported 97% (34/35) of carers agreed to the survey statement “I would recommend this course to a friend.”

#### Completion Rates

Completion rates were inconsistently reported across the papers, with only 5 [52-54,56,57] out of 10 papers [52-61] providing data. Where available, completion rates ranged from 42% [56] to 67% [53,54].

Fair et al [57] analyzed completion rates across 3 iterations of their traumatic brain injury MOOC, finding that educated learners aged 65 years and more and residing in an upper-middle–income country had the highest completion rate (69.5%), followed by learners with personal or family experience with traumatic brain injury (67.9%). However, care providers, including those offering paid, unpaid, or voluntary care to someone living with traumatic brain injury, had the lowest completion rate (60.8%). Participants who were educated, had no previous personal or family experience with traumatic brain injury, and never provided paid care to someone living with a traumatic brain injury completed the course at a rate of 63.4%.

Qualitative findings from Blakemore et al [52] highlighted that their peer-review task, which involved answering questions about another learner’s writing, deterred some participants from completing the course, as it was perceived as too time-consuming. This may have contributed to lower course completion among their cohort.

### Methodological Quality of Included Studies

See Table 4 for ratings from the EPHPP and CASP toolkits. Full appraisal details are available in Multimedia Appendix 2.

**Table 4.** Quality ratings of included studies.

Authors	Study design	EPHPP^a^ rating	CASP^b^ rating
Blakemore et al [Bibr R52][52]	Mixed Methods	Weak	Weak
Borchard et al [Bibr R53][53]	Mixed Methods	Weak	Moderate
Claflin et al [Bibr R54][54]	Quantitative	Weak	N/A^c^
do Canto et al [Bibr R55][55]	Mixed Methods	Weak	Weak
Eccleston et al [Bibr R56][56]	Quantitative	Weak	N/A
Fair et al [Bibr R57][57]	Quantitative	Weak	N/A
Lumini et al [Bibr R58][58]	Quantitative	Moderate	N/A
Rottenberg and Williams [Bibr R59][59]	Mixed Methods	Weak	Strong
Whittingham et al [Bibr R61][61]	Quantitative	Weak	N/A
Whittingham et al [Bibr R60][60]	Quantitative	Strong	N/A

a EPHPP: Effective Public Health Practice Project.

b CASP: Critical Appraisal Skills Program.

c N/A: not applicable.

#### Quantitative Papers

The assessment of quantitative papers (n=6) with the EPHPP tool resulted in 1 paper [60], an RCT design, obtaining a “strong” rating. Lumini et al [58] obtained a “moderate” rating while 4 papers [54,56,57,61] received “weak” ratings. The weak ratings were attributed to two common issues: (1) the individuals selected were unlikely to be representative of the target population, and there was a low participation rate among those selected individuals [54,56,57]; and (2) it was unclear whether outcome assessors were blinded to the intervention or exposure status of participants and if participants were aware of the research question [54,56,57].

#### Mixed Methods Papers

The quantitative elements of all 4 mixed methods papers received “weak” ratings from the EPHPP tool [52,53,55,59]. However, within the “weak” overall score for the quantitative part, Borchard et al [53] demonstrated strength in the reliability and validity of their data collection tools.

The assessment of the qualitative elements of the 4 mixed methods papers yielded 2 “weak” ratings [52,55], 1 “moderate” rating [53], and 1 “strong” rating [59]. However, within the “weak” overall score for the qualitative part, Blakemore et al [52] demonstrated strength in terms of having a clear statement of the research aims, using qualitative methodology appropriately, and producing valuable research. Similarly, do Canto et al [55] had a clear statement of the research aims and used a qualitative approach appropriately.

## Discussion

### Principal Findings

This systematic integrative review identified 10 eligible quantitative or mixed methods papers [52-61] that explored the development, delivery, or evaluation of open online courses for informal carers. While the included papers offer useful insights, the majority ranged from moderate to weak quality. These findings highlight a pressing need for more high-quality research in this area to ensure evidence-based advancements in course design and implementation.

### Quality of Evidence

The methodological limitations observed across papers highlight key areas for improvement. Some common issues included gaps in minimizing participant selection bias, insufficient blinding to research objectives, and inadequate reporting on the validity and reliability of results. Furthermore, 3 [52,53,55] out of 4 mixed methods papers [52,53,55,59] lacked an in-depth presentation of qualitative data and direct participant quotes to substantiate findings, which weakened the strength of evidence. Addressing these methodological shortcomings in future research is essential to enhance the credibility of findings and enable more impactful synthesis on this topic.

### Accessibility and Flexibility

Reflecting the global shift toward flexible learning environments [62], one of the primary strengths of open online courses for informal carers, as highlighted in this review, was their ability to deliver accessible and flexible digital education. The online format allowed learners to engage with the course content at their own pace, from any location, and often at no cost. This flexibility was particularly valuable for informal carers, the majority of whom were women. Globally, women perform 76.2% of unpaid care work [5], dedicating on average 3.2 times more hours to this labor than male peers. For female family carers, open online courses may offer an educational opportunity that can be integrated into their demanding schedules. The ability to revisit content, participate in discussions, and access a variety of multimedia resources were all aspects of open online courses that learners found beneficial.

However, this flexibility also presented challenges. Our review found that the self-paced nature of the courses led to asynchronous participation in discussion boards, which was an issue for learners who progressed through the course at faster speeds. This observation aligns with previous MOOC research indicating that some discussion board posts received no responses [63,64] and most discussion board interactions consisted of single post exchanges rather than ongoing conversations [64]. Similar patterns emerged in this review, suggesting that it can be challenging for open online courses to effectively create a community of learners. This might be considered somewhat problematic given that opportunities for peer connection were identified by many learners as a primary motivation for course enrollment. For instance, Chan et al [65] found that adult carers in the United Kingdom frequently turned to the internet for both information and peer support. One article [53] in this review similarly found that informal carers used discussion boards more frequently than noncarers. The need for peer support is particularly relevant among informal carers, who are at increased risk of loneliness—a condition associated with higher morbidity and mortality risks [66]. For many informal carers, the promise of connecting with others for emotional support is as valuable as the educational content itself [67]. It will be important for future open online course designs to incorporate more synchronous elements, such as live discussions or group activities, which could foster a stronger sense of community among participants.

### Learner Outcomes

The measurement of outcomes in open online courses remains an underdeveloped area within the literature [68]. This gap was evident in our review, where outcome measures were infrequently recorded and, in instances where they were, varied widely across studies, highlighting the need for a more standardized approach. The current findings suggested that open online courses had a generally positive impact on learners, particularly in terms of improving knowledge and caring skills. Most studies that assessed these aspects reported significant gains for learners, underscoring the potential of digital platforms to effectively deliver educational content. The results also align with broader research on online learning, which indicates that digital technologies (eg, mobile apps or web-based platforms) can significantly improve learning outcomes in terms of knowledge acquisition, understanding, and application [69].

However, the impact of open online courses for informal carers on broader psychosocial outcomes was less consistent. While some studies reported increases in carers’ self-confidence and a greater sense of meaning and fulfillment in life [58,60], others found no significant changes in carer well-being [60]. This inconsistency could suggest that existing studies are yet to measure outcomes that fully capture these psychosocial impacts. It is also possible that while open online courses may be effective in knowledge dissemination, they may be less capable of addressing the emotional and psychological needs of carers. Research highlights psychosocial challenges, such as emotional management [70], psychological distress [71], and reduced social connectedness [72], as common unmet needs among informal carers. This presents an opportunity to enhance open online courses by incorporating more participatory and personalized elements, such as real-time discussions and peer support. However, before definitive conclusions can be drawn about the outcomes of open online courses for informal carers, more research is needed to standardize outcome measurements. Standardization will allow for more reliable evaluations and a clearer understanding of how best to support carers through these courses, allowing for more meaningful comparisons across studies.

### Barriers to Participation

The review identified several barriers that potentially limit the effectiveness and accessibility of open online courses for informal carers. Technical difficulties, such as navigation issues and limited access to reliable internet, were frequently cited as challenges by learners. These issues were more marked in different subgroups of carers, including older adults, those living in rural or remote geographical locations, and those who may lack the digital skills or infrastructure needed to fully engage with e-learning platforms [59]. The digital divide has long been a focal point of discussion regarding open online courses. Gameel and Wilkins [73] highlight that MOOC learners from countries with late internet adoption often have lower levels of engagement with information and communication technologies compared to those from countries with earlier adoption. Similarly, studies indicate that older adults, who typically have lower internet usage rates than younger individuals, may struggle to navigate open online courses effectively [74]. Given that a large proportion of informal carers are aged between 50 and 75 years [6], this challenges the assumption that all informal carers on open online courses have similar digital skills. Educators should acknowledge the varying levels of technological competence across demographic groups when designing these courses, especially in the context of the global aging population.

Another important but less frequently discussed barrier relates to language and literacy. Our review identified an absence of papers from non-English–speaking regions despite widespread smartphone and internet use globally, which reflects, in part, our inclusion of only English language studies. Language proficiency and literacy levels significantly influence learners’ ability to engage with course content. United Nations Educational, Scientific and Cultural Organization (UNESCO; 2008) noted that learners performed better when courses were offered in their native language, presenting a challenge for non-native English speakers [75]. While multimedia elements, such as videos, audio, and participatory activities may help address literacy challenges, there is limited evidence on how well these tools are adapted to the diverse linguistic and cultural contexts of learners globally. Without such adaptations, carers from non-English–speaking backgrounds or with lower literacy may face additional hurdles benefiting from these courses. Addressing these issues requires course developers to prioritize language inclusivity and culturally relevant content, alongside varied delivery modes, to improve accessibility and engagement.

Barriers to participation were also increased by carers’ responsibilities. Fair et al [57] found that individuals with paid, unpaid, or voluntary caring responsibilities for someone with traumatic brain injury had the lowest completion rates in their MOOC, while learners with personal or family experience with traumatic brain injury but no caring responsibilities had one of the highest rates of completion. This implies that while a connection to a condition may motivate learners to engage with an open online course, care duties can create significant barriers to participation. Past literature aligns with this finding, showing that carers report that they have unmet needs in terms of time constraints [76-78]. This underscores the need for flexible course designs that better accommodate carers’ schedules and caring responsibilities.

While the courses reviewed generally received positive feedback regarding their user-friendliness, there is a need for enhanced support systems to help learners navigate these barriers. For example, Hoter and Nagar [79] found that older MOOC learners tend to prefer technical support from real people rather than automated responses. In addition, because many MOOC learners benefit from support tailored to their individual learning styles [80,81], offering a variety of support options is essential. This may take the form of topic-organized FAQs, live Q&A sessions, instructional videos, or access to technology advisors and support chatbots.

In addition to technical challenges, the review highlighted social and psychological barriers to learner participation. Some learners expressed discomfort with the public nature of discussion boards, citing privacy concerns as a reason for not fully engaging with peer interactions. This issue points to a broader tension within open online courses between the need to foster community versus the need to safeguard privacy [82]. While open online courses rely on openness and transparency to promote social learning, this openness can sometimes work against participation—especially for informal carers, who often face stigma surrounding caring responsibilities [83]. Research shows that informal carers of individuals with mental health or alcohol-related conditions experience heightened stigma and can feel that their role and the needs of their care recipients are undervalued [65]. This stigma may leave some carers somewhat more reluctant to share their difficulties with others. Many carers welcome the ability to search for information on the web without the pressures of face-to-face contact, which can provide a level of comfort and autonomy not always found in traditional carer support groups [65].

The open nature of open online courses presents challenges for maintaining privacy, but simple adjustments, such as offering learners the option to participate anonymously or use pseudonyms, could help address these concerns. Previous studies on web-based carer support communities have found that measures to vet members can increase interaction [84]. Although vetting is not feasible in open online courses (as they are designed to be accessible to all), even minimal privacy options could encourage higher engagement from learners. Almatrafi and Johri [85] found that approximately 10% of discussion posts in MOOCs were anonymous, with this proportion increasing as courses progressed, suggesting a growing desire for privacy among learners. Anonymity could create a safer environment for informal carers to share their experiences without fear of judgment.

### Limitations

To the best of our knowledge, this is the first systematic integrative review to comprehensively map the landscape of open online courses specifically for informal carers. The insights gained from this review offer guidance for the design and implementation of future open online courses, helping to better tailor these courses to the needs of informal carers. However, several limitations should be considered.

First, a quality assessment of the methodologies used by the selected papers was not conducted to exclude publications, which resulted in incorporating findings from studies with varying methodological rigor. This may have introduced some bias and limited the strength of our conclusions. We attempted to mitigate this by transparently reporting quality assessments (see Table 4 and Multimedia Appendix 2), which allows readers to interpret findings in light of study quality.

Second, the variability in reporting of development, outcome measures, and engagement metrics across the studies made it challenging to draw consistent conclusions about the impact of these courses. This variability was reflected in the moderate-to-weak quality ratings assigned to many of the included studies. While our use of a structured narrative synthesis helped identify recurring themes, the heterogeneity of reporting limited cross-study comparisons.

Third, this review encompassed a broad range of learners, from carers to health care professionals and students, all participating in open online courses designed for informal carers. This participant diversity made it difficult to ascertain how the experiences of different learner groups may have impacted their engagement and outcomes in these courses. Shapiro et al [86] found that MOOC learners with a bachelor’s degree or higher reported more positive experiences than those with a lower educational level, which could pose challenges for learners who are informal carers and often come from diverse educational backgrounds [5]. Furthermore, Jung and Lee [87] found that the amount of time dedicated to learning correlated positively with knowledge growth, suggesting that informal carers may struggle to engage fully with course material due to their caring responsibilities. Supporting this, Teles et al [88] reported that only about 25% of informal carers of individuals with dementia actively sought online resources for personal benefit, such as support services or coping strategies. These findings reveal some of the significant barriers that informal carers face in accessing and using online resources. Combined with language and digital skills challenges discussed earlier [75], these factors highlight the need for future research on open online courses to distinguish between learner groups where possible to better understand the unique experiences of each demographic.

Finally, gray literature was not systematically searched, which may have excluded valuable nonpeer-reviewed evaluations of open online courses. Given that many such courses are developed and delivered outside academic contexts [86], and might have been less likely to have been formally evaluated or published in peer-reviewed journals, this review likely captures only a subset of relevant course evaluations. Future research would benefit from a broader search strategy to better understand the full range of online educational offerings and their effectiveness for informal carers.

### Conclusions

In summary, this systematic integrative review highlights the evolving landscape of open online courses designed for informal carers, identifying both strengths and challenges in course design and delivery. The findings underscore the need for open online courses to enhance accessibility and engagement. By incorporating more peer interaction and synchronous opportunities for connection, open online courses can foster a sense of belonging that is often lacking in online learning environments. Moreover, standardizing outcome measures will allow for more reliable evaluations of these courses in the future. As the demand for informal care continues to grow with the global aging population, the potential of open online courses as an educational tool will only become more significant, necessitating ongoing research and innovation in this space.

## Supplementary material

10.2196/72808 Multimedia Appendix 1 Complete database search strategy.

10.2196/72808 Multimedia Appendix 2 Complete Critical Appraisal Skills Program and Effective Public Health Practice Project quality assessment ratings.

10.2196/72808 Checklist 1 Preferred Reporting Items for Systematic Reviews and Meta-Analyses (2020) checklist.

## Abbreviations

CASP: Critical Appraisal Skills Program
EPHPP: Effective Public Health Practice Project
MOOC: massive open online course
PICO: population, intervention, comparator, outcome
PRISMA: Preferred Reporting Items for Systematic Reviews and Meta-Analyses
RCT: randomized controlled trial
UNESCO: United Nations Educational, Scientific and Cultural Organization
