# Supplementary material for: Open Online Courses for Informal Carers: Systematic Integrative Review
Source: J Med Internet Res. 2025 Aug 28;27:e72808. doi: 10.2196/72808 (PMC12392690; doi:10.2196/72808)
Supplement: Multimedia Appendix 1 [file jmir-v27-e72808-s001.docx]

**Multimedia Appendix 2: Complete Database Search Strategy.**

1. **CINAHL (via EBSCOhost)**

('informal' or 'family' or 'unpaid') and ('carers' or 'caregivers' or 'care' or 'caring') and 'online course'

Limiters: English language, Peer Reviewed

Search modes: Boolean/Phrase

Results: 63 (search rerun on 30 January 2025 with 70 results, 1 new paper identified)

1. **APA PsycINFO (via OVID)**

(('informal' or 'family' or 'unpaid') and ('carers' or 'caregivers' or 'care' or 'caring') and 'online course').mp. [mp=title, abstract, heading word, table of contents, key concepts, original title, tests & measures, mesh word]

Limiters: English language, Peer reviewed journal

Results: 13 (search rerun on 30 January 2025 with 16 results, no new papers)

1. **EMBASE (via OVID)**

(('informal' or 'family' or 'unpaid') and ('carers' or 'caregivers' or 'care' or 'caring') and 'online course').mp. [mp=title, abstract, heading word, drug trade name, original title, device manufacturer, drug manufacturer, device trade name, keyword heading word, floating subheading word, candidate term word]

Limiters: English language

Unable to filter for peer reviewed journals

Results: 61 (search rerun on 30 January 2025 with 67 results, 1 new paper identified)

1. **MEDLINE (via OVID)**

(('informal' or 'family' or 'unpaid') and ('carers' or 'caregivers' or 'care' or 'caring') and 'online course').mp. [mp=title, book title, abstract, original title, name of substance word, subject heading word, floating sub-heading word, keyword heading word, organism supplementary concept word, protocol supplementary concept word, rare disease supplementary concept word, unique identifier, synonyms, population supplementary concept word, anatomy supplementary concept word]

Limiters: English language

Unable to filter for peer reviewed journals

Results: 40 (search rerun on 30 January 2025 with 47 results, 1 new paper identified)
