# Supplementary material for: Open Online Courses for Informal Carers: Systematic Integrative Review
Source: J Med Internet Res. 2025 Aug 28;27:e72808. doi: 10.2196/72808 (PMC12392690; doi:10.2196/72808)
Supplement: Multimedia Appendix 2 [file jmir-v27-e72808-s002.docx]

**Multimedia Appendix 3: Complete CASP and EPHPP Quality Assessment Ratings.**

**Quality appraisal of included studies using the Critical Appraisal Skills Program (CASP) toolkit.**

| **Quality criteria** | **Blakemore et al (2020)** | **Borchard et al (2023)** | **do Canto et al (2023)** | **Rottenberg and Williams (2021)** |
| --- | --- | --- | --- | --- |
| Was there a clear statement of the aims of the research? | Y | Y | Y | Y |
| Is a qualitative methodology appropriate? | Y | Y | Y | Y |
| Was the research design appropriate to address the aims of the research? | C | C | C | C |
| Was the recruitment strategy appropriate to the aims of the research? | N | C | N | Y |
| Was the data collected in a way that addressed the research issue? | N | Y | N | Y |
| Has the relationship between researcher and participants been adequately considered? | N | N | N | N |
| Have ethical issues been taken into consideration? | C | Y | C | N |
| Was the data analysis sufficiently rigorous? | N | N | N | Y |
| Is there a clear statement of findings? | N | C | N | Y |
| How valuable is the research? | Y | Y | C | Y |
| **Total Ranking** | **L** | **M** | **L** | **H** |

**Key**: Y = Yes, C = Can’t tell, N = No

H = High, M = Moderate, L = Low

**Quality appraisal of included studies using the Effective Public Health Practice Project (EPHPP) Quality Assessment Tool.**

| **Quality criteria** | **Blakemore et al (2020)** | **Borchard et al (2023)** | **Claflin et al (2021)** | **do Canto et al (2023)** | **Eccleston et al (2019)** | **Fair et al (2024)** | **Lumini et al (2023)** | **Rottenberg and Williams (2021)** | **Whittingham et al (2020)** | **Whittingham et al (2022)** |
| --- | --- | --- | --- | --- | --- | --- | --- | --- | --- | --- |
| Selection Bias | W | W | W | W | W | W | W | M | M | M |
| Study Design | M | M | M | W | M | M | M | M | S | S |
| Confounders | W | M | S | W | S | S | M | W | W | S |
| Blinding | W | W | W | W | W | W | M | W | M | M |
| Data Collection Methods | W | S | W | S | S | S | S | W | S | S |
| Withdrawals and Dropouts | W | M | M | W | M | M | M | W | M | S |
| Intervention Integrity | M | M | M | M | M | M | M | M | W | M |
| Analyses | M | M | M | M | M | M | M | M | S | S |
| **Global Rating** | **W** | **W** | **W** | **W** | **W** | **W** | **M** | **W** | **W** | **S** |

**Key**: S = Strong, M = Moderate, W = Weak
